# Sub-Micrometer-Scale Mapping of Magnetite Crystals and Sulfur Globules in Magnetotactic Bacteria Using Confocal Raman Micro-Spectrometry

**DOI:** 10.1371/journal.pone.0107356

**Published:** 2014-09-18

**Authors:** Stephan H. K. Eder, Alexander M. Gigler, Marianne Hanzlik, Michael Winklhofer

**Affiliations:** 1 Department of Earth and Environmental Sciences, Ludwig-Maximilians-University Munich, Munich, Germany; 2 Center for NanoScience (CeNS), Munich, Germany; 3 Department of Chemistry, Elektronenmikroskopie, Technical University Munich, Munich, Germany; University Hospital of the Albert-Ludwigs-University Freiburg, Germany

## Abstract

The ferrimagnetic mineral magnetite 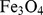 is biomineralized by magnetotactic microorganisms and a diverse range of animals. Here we demonstrate that confocal Raman microscopy can be used to visualize chains of magnetite crystals in magnetotactic bacteria, even though magnetite is a poor Raman scatterer and in bacteria occurs in typical grain sizes of only 35–120 nm, well below the diffraction-limited optical resolution. When using long integration times together with low laser power (<0.25 mW) to prevent laser induced damage of magnetite, we can identify and map magnetite by its characteristic Raman spectrum (303, 535, 665 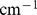) against a large autofluorescence background in our natural magnetotactic bacteria samples. While greigite (cubic 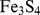; Raman lines of 253 and 351 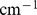) is often found in the *Deltaproteobacteria* class, it is not present in our samples. In intracellular sulfur globules of *Candidatus* Magnetobacterium bavaricum (*Nitrospirae*), we identified the sole presence of cyclo-octasulfur (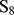: 151, 219, 467 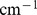), using green (532 nm), red (638 nm) and near-infrared excitation (785 nm). The Raman-spectra of phosphorous-rich intracellular accumulations point to orthophosphate in magnetic vibrios and to polyphosphate in magnetic cocci. Under green excitation, the cell envelopes are dominated by the resonant Raman lines of the heme cofactor of the b or c-type cytochrome, which can be used as a strong marker for label-free live-cell imaging of bacterial cytoplasmic membranes, as well as an indicator for the redox state.

## Introduction

The ferrimagnetic mineral magnetite (stoichiometric formula: 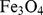) is found as biomineralization product in magnetotactic microorganisms such as bacteria [1], algae [2], protozoans [3], and a diverse range of animals, e.g., molluscs [4], teleost fish [5], [6] and birds [7], [8]. In magnetotactic microorganisms, typically a dozen of intracellular magnetite crystals, the magnetosomes, with 35–120 nm particle size each, are arranged in the form of one or several magnetosome chains, imparting a magnetic dipole moment to the cell body (for recent reviews, see [9]–[11]). The chains are mechanically linked to the cytoplasmic membrane, so that the magnetic torque due to an external magnetic field acting on the chain can be directly transmitted to the cell body, thereby aligning the swimming cell with the external magnetic field. The advantage of swimming along magnetic field lines, a behavior referred to as magnetotaxis, is not fully understood yet, since key aspects of the ecology and metabolism of these microorganisms remain to be elucidated. Similarly, the role of magnetite in animals is only partly understood. It is likely to be involved in mediating a magnetic sense in animals, at least in salmon and homing pigeons, because magnetite found in various nerve tissues of these animals occurs in the magnetic single domain grain size range [5]–[7] just like in magnetic bacteria. In contrast, magnetite in chiton teeth serves as hardening agent [4] and occurs in particles sizes of 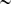 200 nm, above the single domain threshold size [12]. The origin and role of nanoparticulate magnetite (<10 nm) in the pigeon beak [8] and in pathological human brain tissue [13], [14] is unknown.

Since biomineralized magnetite always occurs in grain sizes well below the optical diffraction limit, conventional transmission electron microscopy (TEM) combined with selected-area electron diffraction is the usual method of choice for detection and imaging of intracellular magnetite. Advanced TEM techniques such as off-axis electron holography can be used to gain additional information on the magnetic microstructure of magnetosome chains [15]. With the advent of X-ray zone-plate lenses that limit X-ray spot sizes down to few tens of nm, scanning transmission X-ray microscopy (STXM) allows one to visualize bacterial magnetosome chains and map the magnetic polarity along the chain [16]–[18], albeit at a coarser resolution compared to electron holography. Yet, as a synchrotron-based method, STXM is far from becoming a routine technique. The magnetic dipole pattern due to a magnetosome chain can also be visualized with magnetic force microscopy [19]–[22] and with near-field scanning optical microscopy [23] provided that the chain is close to the surface of the sample, which should be sufficiently smooth to avoid topographic artifacts. Recently, the magnetic stray field distributions due to magnetosome chains were mapped with a resolution of 400 nm using optically detected magnetic resonance [24].

Here we demonstrate that confocal Raman spectroscopy is an effective tool for detecting and mapping intracellular magnetite crystals with grain sizes of typically 100 nm, well below the optical resolution limit. In essence, the effective resolution is enhanced by first acquiring Raman spectra on a grid with mesh width 100 nm and then by filtering the spectra for the specific Raman lines of magnetite. Confocal Raman microscopy has been used to detect biomineralized magnetite in situ [25], [26], but to our knowledge not for mapping the intracellular distribution of magnetite. Confocal laser scanning microscopy in reflectance mode [27] or transmission mode [28] has been applied to image chains of magnetosomes, but relies on additional analytical techniques to determine the chemical composition of the material. Compositional information is important because magnetosomes in the *Deltaproteobacteria* can also be made of the ferrimagnetic sulfospinel greigite (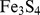) [29]–[38], which compared to magnetite has a 50 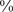 lower saturation magnetization but a higher anisotropy field, which helps stabilize the magnetization of greigite magnetosomes against thermal fluctuations [39].

**Figure 1:**
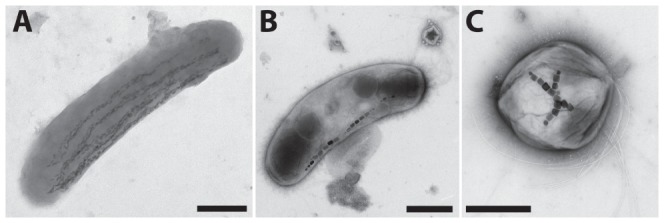
Transmission electron micrographs of three different morphotypes of magnetotactic bacteria extracted from lake sediments. (**A**) *Candidatus* Magnetobacterium bavaricum (MBav), (**B**) magnetotactic vibrio, and (**C**) magnetotactic coccus. (scale-bars are 1 *µ*m).

For Raman-based compositional mapping, we here selected two types of uncultured magnetic bacteria that contain magnetite crystals with typical length dimension 100 nm, *Candidatus* Magnetobacterium bavaricum (MBav, Figure 1A) and uncultured magnetic vibrios (Figure 1B). From magnetotactic cocci (Figure 1C) we later recorded Raman spot-measurements for comparison. All the bacteria were extracted from sediments of lake Chiemsee in Bavaria (Southern Germany) [40]. In the vibrios, the magnetite crystals are arranged in the form of a single-strand magnetosome chain consisting of 10 to 20 crystals of regular morphology with prismatic habit (see Figure 1A), a trait that is only known in magnetic bacteria from the *Alphaproteobacteria* and a single member (strain SS-5) from the *Gammaproteobacteria*, but not from *Deltaproteobacteria* and *Nitrospirae* [41], [42]. In contrast, the large, rod-shaped cells of MBav (Figure 1A) contain up to 1000 magnetite crystals of claw-shaped habit (typical size of 40 nm thick and 100 nm long), arranged in multi-strand bundles of chains along the axis of the cell body [40], [43]–[48]. MBav with its peculiar chain configuration and phylogenetic affiliation to the *Nitrospirae* phylum was long considered an exotic representative of the group of magnetic bacteria, but meanwhile magnetotactic bacteria of the *Nitrospirae* phylum with chain architectures similar to those in MBav have been reported worldwide [49]–[51].

Apart from magnetosome chains, other intracellular accumulations of inorganic compounds in magnetic bacteria may be phosphorous containing granules and/or sulfur globules [52]. Bacteria in general accumulate inorganic phosphorous-compounds in the form of ortho-, poly- or pyrophosphate in intracellular granules [53]. At least three different forms of sulfur 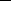 i.e., polysulfanes, cyclo-octasulfur 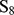, and polythionates 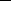 have been suggested to be present in bacterial sulfur globules using X-ray near-edge absorption spectroscopy (XAS) [54]–[57] or Raman spectroscopy [58], [59]. However, as pointed out by George et al. [57], earlier XAS-based evidence of polymeric sulfur in sulfur bacteria may well have been an experimental artifact. 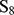 so far has only been identified in the *Proteobacteria* [60], but to our knowledge not in the *Nitrospirae* phylum. In MBav, elemental sulfur has been suggested to be the main constituent of sulfur globules based on their solubility in methanol [44], but the exact allotrope remains unknown. Using the Raman reference spectra of sulfur allotropes compiled by Eckert and Steudel [61], we will here determine the nature of the sulfur allotrope in MBav.

## Results

**Figure 2:**
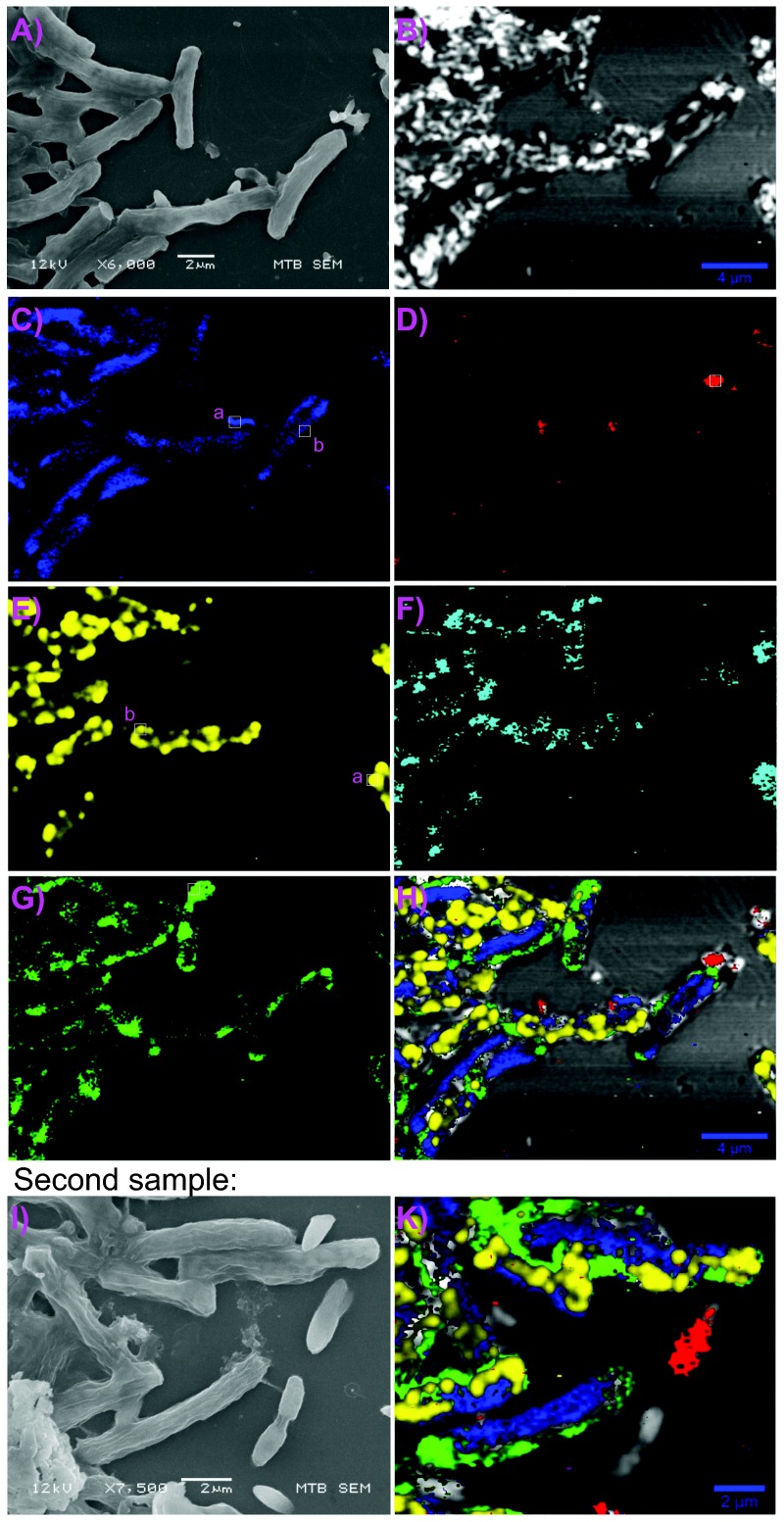
Raman-based compositional mapping of bacteria samples. (532 nm, laser power 0.17 mW) **A)** Scanning-electron micrograph (SEM) showing 6–10 *µ*m long rod-shaped cells of MBav and less elongated (1–3 *µ*m) vibrios. **B)** Confocal reflectance, simulated from scattering intensity at Rayleigh peak. **C)** map of magnetite (303, 535, 665 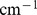), which forms linear structures (magnetosome chains). **D)** map of orthophosphate (1080 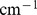, P-O stretching mode), **E)** map of 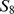 (filter: 151, 219. 467 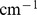), which occurs in the form of intracellular globules, **F)** map of 800 to 950 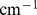 band, see also Figure 3Bi and 3E upper spectrum. **G)** map of 747 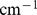 (cytochrome), closely associated with the plasma membrane, **H)** composite map of B, C, D, E, G. **I)** SEM of another sample, with corresponding composite Raman map shown in **K** (532 nm laser, 0.25 mW, same filters and coloring scheme as in H).

**Figure 3:**
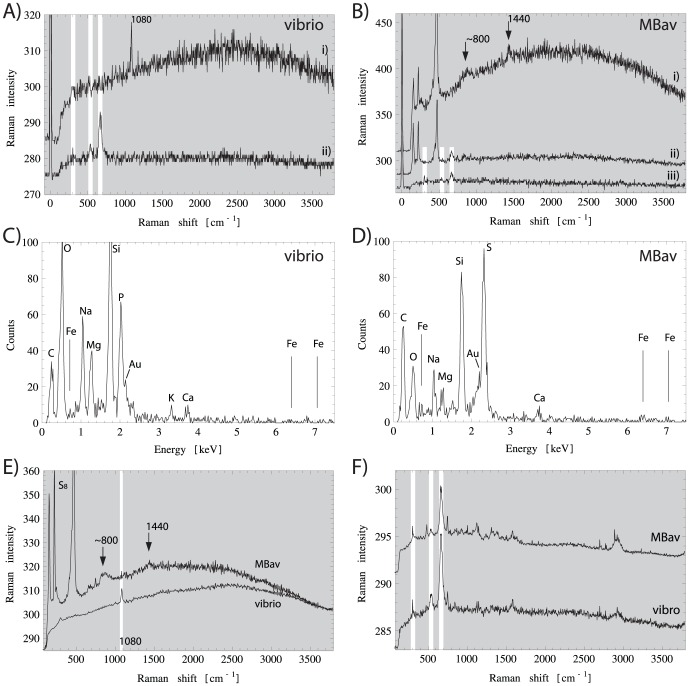
Typical Raman spectra of magnetic bacteria. **A-B)** recorded at a single-pixel (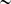
400 nm size), as marked in Figure 2 (white open boxes). **A)** vibrio: (i) intracellular phosphorous reservoir (white box in Figure 2D) with a sharp line at 1080 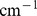, characteristic of orthophosphate. (ii) Magnetosome chain (box 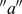 in Figure 2C), with the three characteristic lines of magnetite at 303, 535, and 665 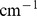 (white bars). **B)** MBav (i) intracellular sulfur globules (box 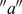 in Figure 2E), dominated by 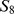 rings (151, 219, 467 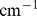); arrow shows broad band (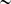
800 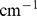) assigned to two-phonon peak of 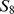. The line at 1440 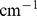 is typical of fatty acids. (ii) Magnetite (white bars) in cells containing also sulfur globules (box 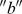 in Figure 2E). (iii) Magnetite in cells without sulfur globules (box 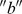 in Figure 2C). **C)** EDX-spectrum of magnetic vibrio, dominated by phosphorous and oxygen. The Si-line is due to the microscope slide, and the Au-line due to the sputtered gold at the surface. **D)** EDX-spectrum of MBav. Sulfur is clearly present, while oxygen is less abundant than in C. **E, F)** Average spectra obtained by averaging over those regions that exclusively produce Raman lines of sulfur in MBav (E, upper graph), of orthophosphate in vibrio (E, lower graph), and of magnetite in MBav (F, upper graph) and in vibrio (F, lower graph).

**Figure 4:**
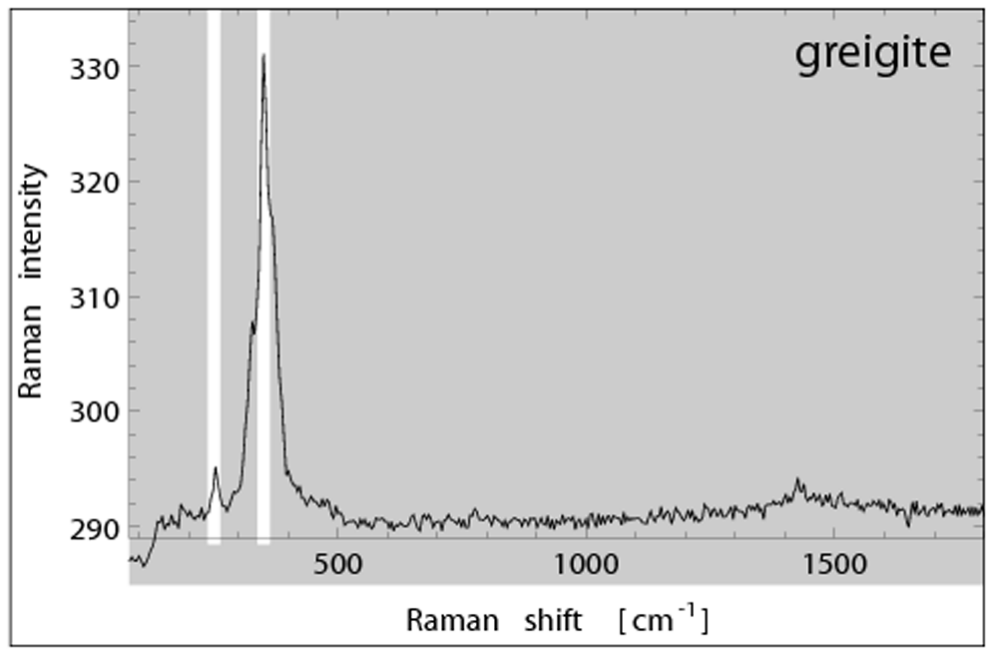
Raman spectrum of a single crystal of greigite (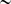
10 *µ*m grain size). The band at 351 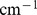 has a satellite at 327 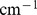 and a shoulder at 367 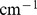. Another line is located at 253 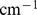. Note the absence of lines at wavenumbers> 500 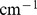, particularly in the 660–670 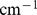 range, where magnetite and hematite are active. The 190 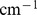 line is just not detectable. The spectrum is consistent with observations from literature (c.f. Table 2 and [96]).

**Figure 5:**
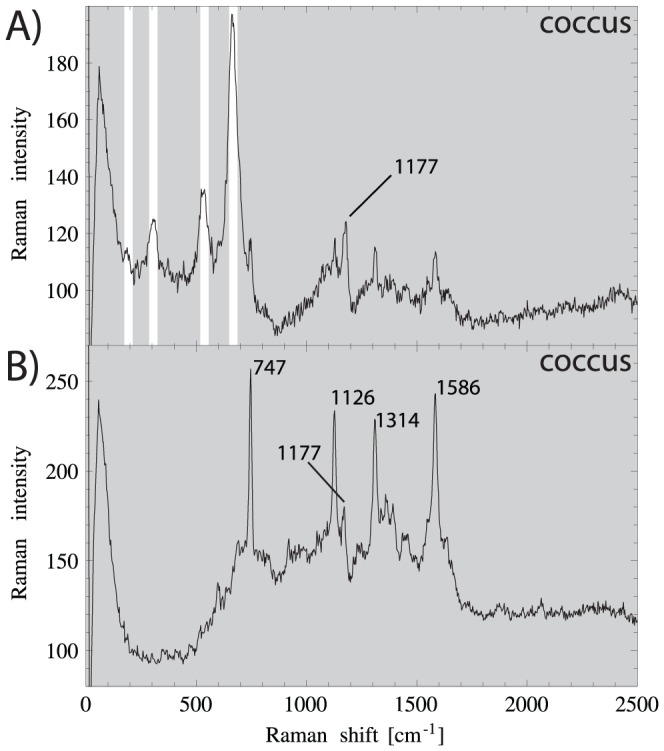
Single Raman spectra of a magnetotactic coccus, taken at two different spots with otherwise identical settings. **A)** Well expressed magnetite lines at 190, 303, 535, 665 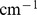 (white bars). **B)** lines of heme group (most likely of reduced cytochrome c) at 747, 1126, 1314, 1586 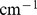. The line 1177 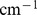 is present in A) and B) similarly strong, and therefore is not assigned to the heme group, but to polyphosphate (Table 2).

In our sample of magnetotactic bacteria (Figure 2) five typical Raman spectra were picked from single voxels in Mbav and vibrio cells (Figure 3A and B). While the background in the single-voxel spectra is dominated by autofluorescence from organic compounds, one can see a number of distinct lines superimposed. When stacking single-voxel spectra (see Methods), the relevant lines become more dominant (Figure 3E-F). The set of three lines at 303, 535, and 665 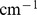 corresponds to stoichiometric 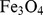 ([62], c.f. Table 1), while greigite (cubic 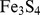), as often observed in magnetotactic bacteria from the *Deltaproteobacteria* taxon, would produce lines at 253 and 351 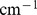 (see Figure 4 and Table 2). Another magnetite line at 193 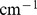 which usually is weak [62], [63], appears in the spectrum of a coccus (Figure 5A and Table 2), but not in the one of Mbav or vibrio. On the compositional map for magnetite (Figure 2C), one can clearly recognize linear structures of magnetite in all MBav cells and in some vibrio cells (compare Figure 2C feature 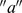 with Figure 2A). However, magnetite chains cannot be detected in all vibroid cells in this particular focal plane, which we ascribe to the small focal depth in the confocal set up: magnetite chains lying above or below the focal plane would be missed. In contrast, MBav contains between two and five multi-strand-chains of magnetosomes, so that the probability of observing a strand of magnetosomes in the focal plane is higher.

**Table 1:** Characteristic Raman lines of iron-oxides and iron-sulfides, compiled from literature.

Magnetite (Fe_3_O_4_)				Maghemite (γ -Fe_2_O_3_)		
[62]	[92]	[91]	[90]	[92]	[91]	[93]
193m	193w			350w	350w	360
**306m**	**306m**	**310m**	302m	500w	512w	**500**(wide)
			513m		**665s**	
**538m**	**538m**	**536m**	533m	700w	**730s**	**700**(wide)
**668s**	**668s**	**667s**	663m		**1330s**	

Hematite (*α*-Fe_2_O_3_)				Greigite (Fe_3_S_4_)	Pyrite (FeS_2_)
[62]	[92]	[91]	[90]	[92]	[91]
**226m**	**225s**	**225m**	225s	139w	351m
**245m**	245m			188m	**386s**
292w	**291–300m**	**291m**	292s	**250m**	**443s**
299w				**351s**	
**411vs**	412m	**411m**	408s	**366sh**	
497w	500w				
612m	611w		613m		
**659vs**	661w	**671s**			
817w	817w				
1049w					
1103w					
**(1320vs)**	**1321vs**	**1321vs**			
	(synthetic)	(rustle rouge)			

**Table 2:** Raman lines identified in our bacteria samples, and in synthetic reference samples.

MBav				
Magnetite	S_8_	S_8_	CH_2_ fatty	Cytochrome
		crystalline	acids	C (II)
Fig. 3B,F	Fig. 3B,E	Fig. 3B,E	Fig. 3B,E	Fig. 3B,F
**303s**	**151vs**	**800–950**	**1440m**	**750s**
**535m**	**219vs**	(wide)		
**665vs**	**467vs**			

Vibrio			Cocci		
Magnetite	Orthophosphate	Cytochrome	Magnetite	Cytochrome	Polyphosphate
		C (II)		C (II)	
Fig. 3A,F	Fig. 3A,E	Fig. 3F	Fig. 5A	Fig. 5B	Fig. 5A,B
**303s**	**1080s**	**750s**	193m	**747vs**	**1177s**
**535s**			**303s**	**1126vs**	
**665vs**			**535s**	**1314vs**	
			**665vs**	**1586vs**	

Synthetic greigite	Sodium thiosulfate		Sodium polyphosphate		Sodium phosphate	
Fig. 4	**		**		**	
**253m**	**72s**	**448vs**	221w	730sh	135w	532sh
327sh	93sh	533w	308sh	883w	226w	**870vs**
**351vs**	**125s**	556w	330m	950w	358m	**922vs**
**367sh**	150w	**669vs**	388sh	1008w	420m	**998s**
*	216w	**1000vs**	457w	**1158vs**	499m	1075w
	**322s**	**1135s**	511m	1261m	522sh	1175w
	360w	**1155s**	**684s**		541m	1288w

* Note the absence of lines at wavenumbers> 500 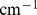.

** see also supplement Figures S1 to S3.

The energy dispersive X-ray (EDX) spectrum of the vibroid bacterium shows a pronounced phosphorous peak (at 2.0 keV, see Figure 3C). Phosphorous in bacteria is typically stored in acidocalcisomes in the form of orthophosphate (Pi), pyrophosphate (PPi), or polyphosphate (poly-P) [53] (see Table 3 for their Raman shifts). The characteristic Raman shifts of neither poly-P (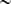
700 and 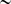
1170 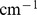, [64], [65]) nor PPi (1022 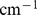 [66]) (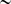
1050 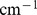 [67], [68]) are detectable in the Raman spectrum of the vibroid bacterium (Figure 3A and 3E). However, we can identify a sharp peak at 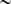
1080 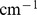, which we assign to the P = O symmetric stretching mode of PO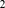 [69], [70]. The 874 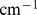 line due to symmetric stretching of P(OH)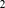 is not detectable. Thus, the main constituent of the intracellular phosphorous reservoirs in the vibrios (Figure 2D) appears to be orthophosphate, the most stable phosphate.

**Table 3:** Candidate phosphorous compounds in bacterial inclusions (according to DoCampo [53]) and characteristic Raman lines for inorganic equivalents compiled from literature.

Pi	poly-P	Na-PPi	Ca-PPi
[69], [70]	[64], [65]	[66]	[67]
380m	**700s**	738w	**353s**
514m	**1170vs**	**1122s**	363sh
**874s** (sym. Stretch of P(OH)2)	2940m	1102w	440w
			486–620w (several)
940w			741sh
**1075s** (P = O stretch of PO2)			755s
			919w
1150w			**1052vs**
			1084w
			1117m
			1125m
			1186m

Pi  =  orthophosphate; poly-P  =  polyphosphate; PPi  =  pyrophosphate.

A distinct sulfur peak (2.3 keV) is seen in the EDX spectrum of MBav, resulting from intracellular sulfur globules. We can identify the chemical nature of sulfur in these globules from the Raman spectrum (Figure 3B), which shows a set of three lines at 151, 219, and 467 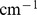 (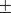 2 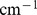). These can be uniquely attributed to elemental oct-atomic sulfur, 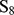 (85, 152, 216, 470 , [71], c.f. Table 4, but note that 85 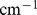 is not detectable with our Rayleigh filter). The two lines at 151 and 219 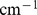 represent bending modes characteristic of the S_8_-ring. Polymeric sulfur, reported for purple and green sulfur bacteria [55] (however, see [57]) and also suggested to be the form of sulfur in MBav [45], would have lines at 260, 275, 425, and 460 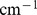 (see [61] and Table 4), which however were not detectable in our samples. Characteristic lines of other elemental sulfur allotropes from S_6_ to S_20_ (compiled in [61] and Table 4) were not detectable either. The asymmetric line at 467 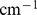 represents a merger of S-S stretching modes at different Raman shifts within the 460–480 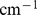 band [61]. A small but distinct shoulder occurs at 415 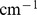, corresponding to a stretching mode that is Raman inactive in molecular 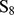 [61], but active in orthorhombic sulfur crystals (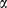
–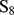). This suggests that 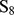 in MBav occurs in condensed structures rather than in the form of isolated molecules. These structures have poor crystallinity as indicated by the width of the asymmetric 467 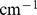 line, which is typical of glassy 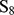, while 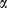
–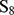 crystals would produce a distinct line at 434 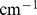 of medium intensity (see [58] and Table 4). As can be seen on the 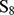 map (Figure 2E) some cells of MBav contain up to 20 intracellular 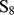 globules of 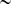
0.5 *µ*m diameter, whereas other cells do not bear detectable amounts of 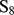. Round reflective features in the Rayleigh map (Figure 2B, which simulates confocal reflectance qualitatively) are co-localized with 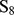 inclusions, while reflective features with irregular morphology do not correspond to sulfur inclusions but rather to unspecific surface features such as sediment or salt particles.

**Table 4:** Characteristic Raman lines of sulfur allotropes, compiled from literature.

S_6_	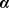 – 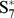	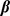 – 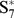	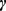 – 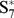	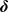 – 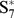	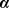 – 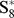	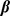 –S_8_
[61]	[61]	[61]	[61]	[61]	[97]	[61]
106w	145m	146m	143m	147w	**150–161vs**	**151s**
**202s**	150w	**155s**	**151s**	150w	182–199w	**153s**
**262s**	**157s**	182m	159w	155m	**217–221vs**	**156s**
**448s**	180m	185m	**178s**	185m	236–239w	195w
**471vs**	185m	**239s**	199w	201w	249–253m	217m
	**239s**	270m	237m	**239s**	419–420w	**221vs**
	241sh	285m	**242vs**	270w	434–442w	243w
	270m	358m	274m	285w	468–473m	249w
	285m	**362s**	285w	358m	**472–474s**	440m
	**355s**	395m	292w	363m	**477–478vs**	467m
	**400s**	**400vs**	**364s**	396m		**272s**
	420w	420w	**400s**	**400vs**		**474s**
	459m	459m	420w	419w	(polarization dependent shifts)	
	**481vs**	**480vs**	459m	460w		
	514m	511m	**481s**	**480s**		
	518s	**518s**	510m	510w		
	530w	528w	523w	518m		
				527w		
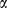 –S_9_	S_10_	S_6_S_10_	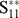	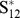	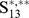	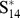
[61]	[61]	[61]	[61]	[61]	[61]	[61]
100m	100m	103m	**176s**	**127vs**	100s	122w
104s	131m	136w	**571vs**	177/179m	**135s**	**128s**
111m	155w	145w		238/245m	**175s**	153m
117m	178m	172m		**289m**	205m	163m
151m	231w	201m		**449s**	280m	177w
155m	243m	207m		460vs	**460vs**	189w
161m	246sh	225w		475w		198m
181m	255w	228w				212w
**188vs**	403w	240m				234m
215m	425w	249w				243w
222m	466sh	265w				252w
245m	**469s**	272m				270w
256m	481sh	408w				444w
297m	487m	428m				447w
416w	495w	451m				453sh
**436s**		455sh				**460s**
442m		461sh				462sh
**454vs**		466m				468m
463m		479s				474wm
477m		489w				483w
485w						
endo- 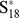	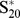	poly- 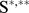	S_8_	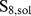	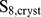	
[61]	[61]	[61]	[58]	[71]	[71]	
**130s**	**131vs**	275w			114w	
156m	248m	260w	**154vs**	**152s**	**152s**	
**165vs**	267m	425w	188w	184w	184w	
230s	**464vs**	**460vs**	**220vs**	**218s**	**216s**	
**450s**			248w	248w	243w	
**465vs**				299w		
**476vs**				334w		
			439w	437w	434m	
			**474vs**	**475s**	**470s**	
				591w		
					**800–950**	
					(twophonon see [61])	
				(in solution)	(crystalline)	

* observed at 300 K or less;

** only the most prominent lines were taken from printed graphs (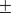
5 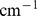)

**Raman line intensity:** w  =  weak; m  =  medium; s  =  strong; vs  =

very strong; sh  =  shoulder of an adjoining line.

Besides the Raman active lines of 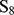, the MBav spectrum (Figure 3B) shows a number of additional lines in the range 800 to 950 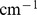 (Figure 3B, i), which have been reported for crystalline 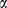
–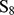 [61] and assigned to combinations of stretching vibrations (two-phonon processes). These are generally much weaker than the characteristic 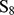 lines. The assignment of the two-phonon band to crystalline 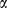
–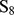 is not unique because some organic groups have vibrational modes in this wavenumber range, too (e.g., [72], [73] or in Table 5). Therefore, to find out if the broad band is associated with sulfur at all, we tested for co-localization on the respective compositional maps (Figure 2E and F). We found the broad band to be always associated with 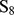 and suggest it is the two-phonon band of sulfur. The intensity of the two-phonon band is significantly weaker than that of 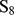.

**Table 5:** Characteristic Raman lines of relevant organic compounds or functional groups (compiled from literature).

Cyto-chrome	Cyto-chrome	Fatty acids	Phospho-lipid	Fd	Ado	RPP
C (III)	C (II)					
[75]	[75]	[73], [ 79]–[82]	[80], [ 99], [ 100]	[101]	[101]	[101]
**757w**	**752vs**	**1063s** *	**715–746**	**282s**	**291s**	290m
1125m	**1128s**	**1130s** *	plus fatty acid peaks	329sh	317w	313w
1170m	1173m	1300s**		**339s**	329w	**336s**
1315m	**1313s**	1440s***		367m	341sh	352sh
**1561s**	**1489s**			395m	**349s**	**387s**
1583m	**1580s**			426m	393w	578m
**1635s**	1617w			563w	421w	641m
				622w	**581s**	675m
				650sh	628sh	736m
				675m	641m	774m
				760w	683m	813m
				794w	741w	
					786m	
					814w	
					868w	

These can be observed also in eukaryotic cells (c.f. [85], [ 98]). Only the most prominent lines of fatty acids (or lipids) are listed. The defining phospholipid line is due to the symmetric diester O-P-O stretching mode of the polar head group and its precise wavenumber depends on various factors (see [100]); for the non-polar tail, see fatty acid lines in the column to the left.

Fd  =  Ferredoxin; Ado  =  Adrenodoxin; RPP  =  Red Paramagnetic Protein

**Figure 6:**
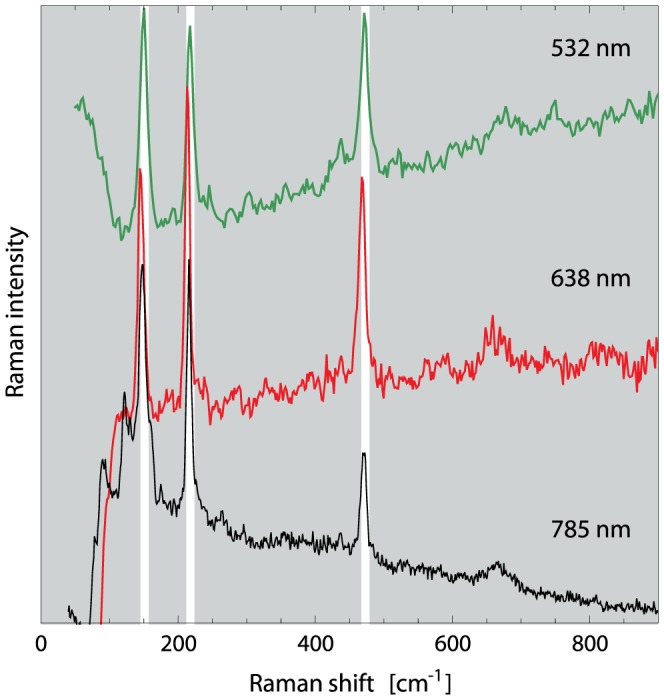
Raman spectrum of a sulfur inclusion in MBav, recorded at three different excitation wavelengths. The characteristic lines of 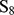 (indicated by the white bars) appear consistently.

*CH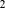 bending/scissoring; **τ-(CH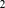) twist; ***skeletal trans C–C stretch.


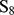
 has an indirect band gap of 2.61 eV [61], that is, light of wavelenghts 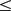 476 nm can be expected to excite resonance Raman scattering. In contrast, the green laser light we used (532 nm, 2.34 eV) should not produce electronic excitations and therefore the 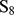 related lines in our Mbav spectra should not be overemphasized due to resonance Raman scattering. To test for resonance effects, we also acquired Raman spectra with yet lower photon energies (red: 638 nm, 1.95 eV; and near infrared/N-IR: 785 nm, 1.58 eV). Figure 6 shows that the three major peaks due to 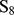 are similarly expressed under all illuminations, which rules out resonance scattering under the green light condition. Due to the different filters used for blocking the Rayleigh band, the characteristic 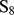 line at 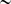
90 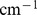 is visible under N-IR, but not under shorter wavelength excitation (Figure 6). Also with the N-IR settings, a peak at 120 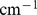 becomes apparent, which is not known for elemental sulfur allotropes, but for oxidized magnetite and maghemite, respectively [74].

Last, a number of robust lines (747, 1126, 1312, 1586 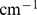, Figure 3F) are spatially associated with each other and concentrated near the cell envelope (Figure 2G). These lines appear dominantly also in the single spectrum of an uncultivated magnetotactic coccus (Figure 5B) and are indistinguishable from those reported by Adar [75] (obtained with the same excitation wavelength as here) for reduced cytochrome c, an electron carrier in the periplasmatic space. This interpretation is consistent with the observation of the cytochrome Raman lines near the cell envelope.

## Discussion

Magnetite is a poor Raman scatterer, and, because of its broad-band absorption characteristics (black color), it readily undergoes laser-induced transformation to maghemite and eventually to hematite [63]. The risk of oxidative alteration is potentially higher for a high surface-to-volume ratio, which is especially the case for sub-100 nm crystals as those in magnetotactic bacteria. Therefore, it is critical to adjust the laser power and scanning settings such that a good compromise between signal strength and alteration level is obtained. With the laser power adjusted to lowest possible intensity level (170–250 *µ*W), the magnetosomes in our samples of MBav, vibrios, and cocci showed the Raman lines characteristic of magnetite, but not of its oxidation products. Therefore our settings prove adequate for studying the mineralogical nature of intracellular and extracellular nano-particles made of iron-oxides, without damaging or affecting the initial composition.

It is noteworthy that magnetosomes can be detected in the Raman confocal map, even though their particle sizes (35–120 nm) are far below the optical detection limit (horizontal resolution: 0.61⋅*λ* NA  =  0.36 *µ*m) and hence not resolvable in the reflectance confocal image (derived from the Rayleigh peak). This virtually enhanced resolution is achieved by processing the acquired spectra, where only those Raman lines are selected that are characteristic of magnetite (303, 535, 665 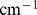). This wavelength filter applied to all pixels scanned then acts as a spatial filter for magnetite, which produces the contrast. To take advantage of the enhanced resolution, the step size for scanning needs to be significantly smaller than the optical resolution limit and here was chosen as 0.1 *µ*m. This allows one to locate the position within a resolvable voxel where a specific material occurs with the highest probability. Although the exact shape and size of the related object cannot be reconstructed, one can assume that its horizontal dimensions are below the optical resolution limit. The Raman map (Figure 2C) clearly demonstrates the arrangement of magnetite particles in the form of one or, as in case of MBav, in the form of multiple chains, consistent with the transmission electron micrographs of Hanzlik et al. [45].

The sulfur globules in the compositional map (Figure 2E) appear as round intracellular structures in MBav. It was known before that MBav accumulates sulfur in intracellular globules, presumably for energy storage [44], [47], [48], but not in what chemical form. Here, we have shown that the enriched sulfur species is elemental sulfur in an S_8_-ring configuration. The two-phonon effect is co-localized with the sulfur globules (see map 2E and 2F), which indicates strong interactions between the atoms of different S_8_-rings. This suggests a dense accumulation of S_8_-rings. Therefore, it is unlikely that material other than 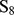 resides in the globules, which is why they can be considered as pure sulfur storages. The cells can use sulfur from these storages to gain energy by oxidation to sulfate (which requires swimming upward from the anoxic sediment where sulfur is collected). Thus, as suggested by Jogler et al. [47], the presence or absence of stored sulfur in individual MBav could quantitatively indicate the energy reserves of the cell (some individual cells of MBav contain 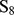, others do not; see Figure 2E).

The source of sulfur for the sulfur globules in MBav is not known yet because MBav is not available in culture. In most bacteria with sulfur inclusions, the sulfur substrate is 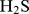 or thiosulfate [60]. Cells of cultivated marine magnetotactic bacteria from the *Alphaproteobacteria* group produce sulfur globules when 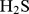 is added to the growth medium (e.g. [76]). For MBav, which is a freshwater bacterium, Spring et al. [44] proposed 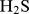 as a substrate, but did not detect sulfide (detection limit 0.01 mM) in the depth zone where cells of MBav occurred. Although small amounts of sulfide cannot be ruled out in these microcosms, the sulfur source of MBav could also be thiosulfate, because MBav was found to have a RuBisCO-like protein that in a green sulfur bacterium is involved in some aspect of thiosulfate oxidation [47]. Otherwise RuBisCO could be involved in autotrophic 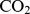 fixation [76].

The co-occurrence of elemental sulfur and magnetite within individual cells of MBav is surprising from the point of view of inorganic equilibrium chemistry, because the stability fields of the two minerals are separated from each other by the stability field of an iron sulfide mineral such as 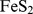 (pyrite) (see figure 7.19 of [77]) or some 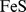 phase (see [78]). The magnetite and sulfur signals (Maps C and E in Figure 2) are often found to co-occur in the same voxel analyzed, but the compounds are enclosed in membrane vesicles each [47] and therefore are spatially separated, which makes chemical reactions between these unlikely to happen. The co-occurrence of elemental sulfur and magnetite within a cell has also been reported in magnetotactic bacteria from the *Alphaproteobacteria* group (e.g. [57]), which keep growing magnetite even when 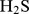 is added to the growth medium (e.g., [76]). In contrast, some *Deltaproteobacteria* produce intracellular greigite crystals (magnetosomes) under high 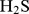 concentrations (> 0.3 mM), but magnetite crystals under low 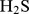 conditions (<0.3 mM) [50].

Spatially associated with the 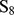 inclusions in MBav, we found the 1440 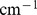 Raman line (Fig. 2B), which most likely represents the 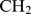 scissoring mode, which is usually pronounced in fatty acids (see Table 5, and [79]–[82]). Sulfur globules, however, have envelopes consisting solely of proteins, not lipids (see [83] for a review), at least in the case of *Proteobacteria*. For this member of the *Nitrospira*, we cannot determine characteristic Raman lines of specific amino acids (e.g. [73]) in the spectra containing the 1440 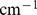 line, yet the CH_2_-related peak would be consistent with side chains of most proteinogenic amino acids.

The set of four lines 746, 1126, 1312 and 1586 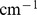 in our coccoid magnetic bacteria (Figure 5) matches the published reference spectra of reduced cytochrome c [75], [84], [85], a heme-group-based perisplasmatic electron transporter involved in the respiratory chain of organisms. The heme group shows a series of strong Raman lines when probed with laser light in the wavelength range 500 nm to 530 nm [84], in which case the first electronic transition (Q transition) of porphyrin is excited so that resonant Raman (RR) scattering occurs. In the reduced state of cytochrome c (i.e., ferrous Fe), the pyrrole breathing mode 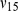 (752 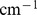) is the most pronounced line ([75] and Table 5), which is also what we observe in the coccus (Figure 5). Cytochrome b and cytochrome c have similar RR spectra at 532 nm [86] and at 569 nm [87], but not at 515 nm [87], which would allow better discrimination between the two heme-proteins. A newly discovered family of c-type cytochrome referred to as magnetochrome, which has been exclusively found in magnetic bacteria [88], is likely to produce similarly strong RR lines as cytochrome c, due to similar UV-Vis absorption spectra. Magnetochrome, which as an iron oxidase controls the proper stoichiometry of magnetite [88], is probably incorporated in the magnetosome membrane, and therefore not periplasmatic as opposed to cytochrome c. Since the magnetosome chains in our bacteria are close to the cytoplasmic membrane [45], we cannot spatially separate signals from magnetochrome and cytochrome c. The presence of reduced cytochrome c, while exposing the cells to oxygen, suggests that cytochrome c has not transferred its electrons onto cytochrome c-oxidase, the terminal electron acceptor of the aerobic respiratory chain. This is probably due to a break down of the proton gradient during cell death while the sample was drying. From a more general perspective, cytochrome c occurs in the periplasmatic space between the two lipid bilayer membranes in prokaryotes, e.g. in bacteria, or in eukaryotic cells, e.g. endosymbionts (mitochondria, chloroplasts). Therefore the Raman cytochrome signal defines the outlines of prokaryotes and it can be used to specifically map intact mitochondria and also to observe apoptotic cells that release cytochrome c into the cytosol [85]. For excitation of cytochrome, lasers tuned to the specific absorption of the heme group can be used at very low laser power to provide a sufficient signal from cytochromes due to RR scattering. This set up drastically reduces damage due to laser-induced heating and therefore is even suitable for examining living cells. This could be used to define for example the cell density on a surface or in a biofilm.

We have shown Raman microspectroscopy to be a powerful tool for the detection and mapping of intracellular sub-100 nm magnetite crystals, even though magnetite is a poor Raman scatterer and cells have a large autofluorescence background. The strength of Raman microscopy is the compound-specific filtering (by selecting the specific Raman lines) that allows one to detect and map objects with sizes below the optical detection limit. Using very low laser power, the excited volume can be kept small and the partial volume effect be maximized, which leads to a high spatial resolution in the wavenumber-filtered maps. With regard to magnetic biominerals, Raman spectroscopy is well suited to distinguish between magnetite and greigite without requiring electron microscopic investigations. Low laser power Raman spectroscopy is also highly suitable for detection and mapping of bacterial sulfur inclusions, where excess heat would cause chemical transformations and therefore experimental artifacts.

## Materials and Methods

Magnetotactic bacteria were extracted from sediments that had been collected earlier from Lake Chiemsee (see [40] for sampling location and [89] for procedure). No permission was required for taking mud samples from Lake Chiemsee. The study does not involve endangered or protected species.

For purification, a drop of sediment is placed onto a microscope slide next to a drop of water. An external magnetic field is used to guide the magnetotactic bacteria from the sediment to the edge of the clear water drop, where the cells accumulate. For transmission electron microscopy (TEM) cells were pipetted off the drop, placed on a carbon coated copper grid and dried on air. For Raman microscopy and scanning electron microscopy (SEM) the cells were dried directly on the microscope slide. Cells were not prepared with biological fixatives in order to avoid additional fluorescence, which reduces the efficiency of Raman detection of inorganic intracellular compounds. The preparation of the samples was done immediately before the Raman analysis.

Confocal Raman imaging was performed with an alpha 300R (WITec GmbH, Ulm, Germany). The excitation wavelength was 532 nm (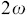-Nd:YAG solid state laser). The laser power was adjusted to very low values (0.17 mW to 0.25 mW) in order to prevent oxidization of the magnetosomes from magnetite to hematite (e.g. [62], [63], [90]–[93]) and to preserve the molecular structure of sulfur [58]. A high numerical aperture (NA = 0.9, 100x) air objective was used in order to efficiently collect scattered light from the measurement spots.

For confocal imaging and compositional mapping, the core of a multimode fiber served as a pinhole with diameter 50 *µ*m, leading to an axial resolution of 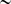 1 *µ*m and a voxel volume of about 1 *µ*m^3^. The piezo-controlled scanning stage carrying the sample was stepped in intervals of 0.1*µ*m in both x- and y-direction. At each confocal point, a Raman spectrum was acquired for 10 sec (10 acquisitions with 1 sec duration each), spanning the wavenumber range 0–3800 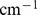, with a grating of 600 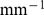. With the low laser power used, such long acquisition times are necessary to identify characteristic peaks of inorganic inclusions against the highly fluorescent background due to the organic material. The Rayleigh peak region (0–100 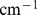) was strongly suppressed with a low-pass filter. The spectrometer was calibrated using a Si-wafer (520 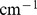). Spikes due to cosmic rays were removed using the de-spiking option in the software. From characteristic Raman lines (and combinations thereof) identified in the measured spectra, we produced compositional maps showing the intensity of these lines at each pixel after background subtraction. On the basis of a threshold-intensity criterion, the intensity-encoded color brightness was adjusted such that only meaningful occurrences of a particular compound are depicted, while all non-meaningful occurrences remain black. Average spectra were obtained by first selecting a specific set of lines, and then by averaging the spectra over those regions that exclusively contain this set of lines. This way, the signal-to-noise ratio for the lines of interest can be enhanced.

An XploRA ONE confocal Raman microscope (Horiba Scientific, Kyoto, Japan), equipped with 532 nm, 638 nm, and 785 nm lasers, was used to acquire Raman point spectra on magnetic coccus bacteria (532 nm). It was also used to study excitation-wavelength dependence of Raman spectra in the sulfur inclusions of MBav, and to obtain the reference spectra of sodium thiosulfate (Sigma no. 72049), sodium polyphosphate (Riedel-de Haën no. 04267) and sodium phosphate (Sigma no. S8282).

For scanning-electron microscopy (performed after Raman analysis) with a JSM5900LV (JEOL, Japan), bacteria samples were plasma coated with a few atomic layers of gold. An acceleration voltage of 12 kV was chosen for SEM imaging. Elemental analysis was done by energy-dispersive X-ray (EDX) analysis at 12 kV (Röntec, Germany).

TEM micrographs of the separately prepared bacteria were taken with a JEM2011 (JEOL, Japan) operated at 120 kV.

In order to obtain a clean Raman reference spectrum for greigite, we hydrothermally synthesized greigite from iron(III)-chloride, thiourea, and formic acid, closely following the protocol by Tang et al. ([94]; see also [95]), and recorded with the low laser-power setup (0.2 mW, 532 nm) from a single greigite crystal with 10 accumulations over 5 sec integration time each. The Raman spectrum obtained (Figure 4) is characterized by a distinct line at 253 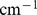 and a broad band peaking at 351 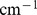 with shoulders at 327 and 367 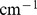. This is consistent with the Raman spectrum obtained on a greigite powder sample with a 633 nm laser line and a 0.2 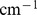 grating [96]. Notable is the absence of lines above 500 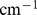, which has not been reported in the literature yet. The dominant line for iron oxides which usually is around 660–670 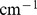 [63] is not present in the greigite spectrum. Since sulfur is heavier than oxygen, and the force constants for the bonds involving sulfur are smaller than those for oxygen, the vibration frequencies are lower. Lines of residual sulfur compounds from the synthesis or of possible greigite decomposition products were not detectable, which indicates the purity of the sample. The greigite lines (253 and 351 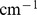) are right in between the characteristic lines of magnetite (303 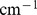 and 535 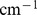) and therefore the two minerals are well distinguishable in Raman spectroscopy. However, we found no evidence for greigite in the bacteria samples studied here.

## Supporting Information

Figure S1 **Raman spectra of sodium thiosulfate under three different excitation wavelengths.**
(PDF)

Figure S2 **Raman spectra of sodium phosphate under three different excitation wavelengths.**
(PDF)

Figure S3 **Raman spectra of sodium polyphosphate under three different excitation wavelengths.**
(PDF)
