# Supplementary figures and images for: Sub-Micrometer-Scale Mapping of Magnetite Crystals and Sulfur Globules in Magnetotactic Bacteria Using Confocal Raman Micro-Spectrometry
Source: PLoS One. 2014 Sep 18;9(9):e107356. doi: 10.1371/journal.pone.0107356 (PMC4169400; doi:10.1371/journal.pone.0107356)

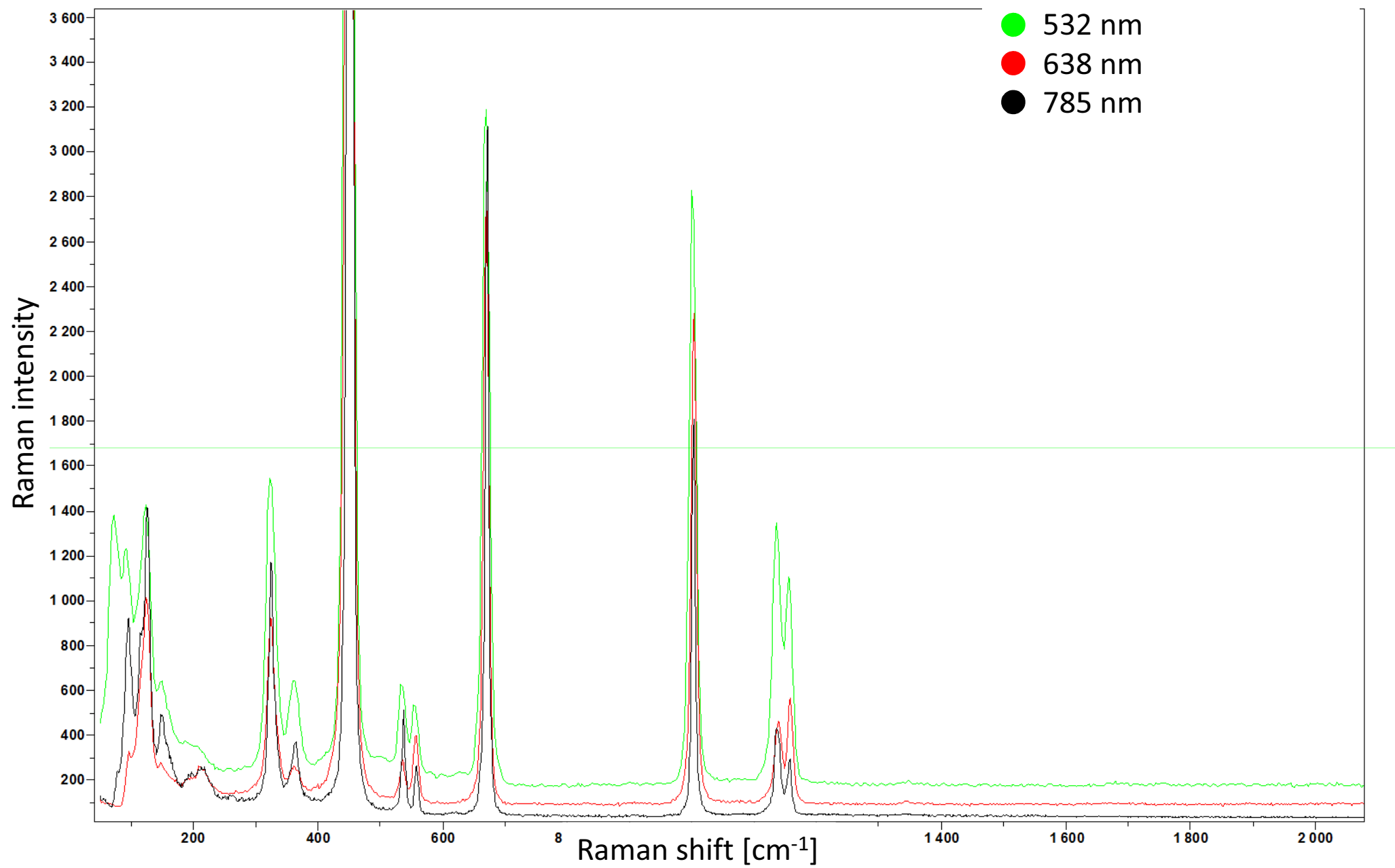

Supplement: Figure S1 — Raman spectra of sodium thiosulfate under three different excitation wavelengths. (PDF) [file pone.0107356.s001.pdf]

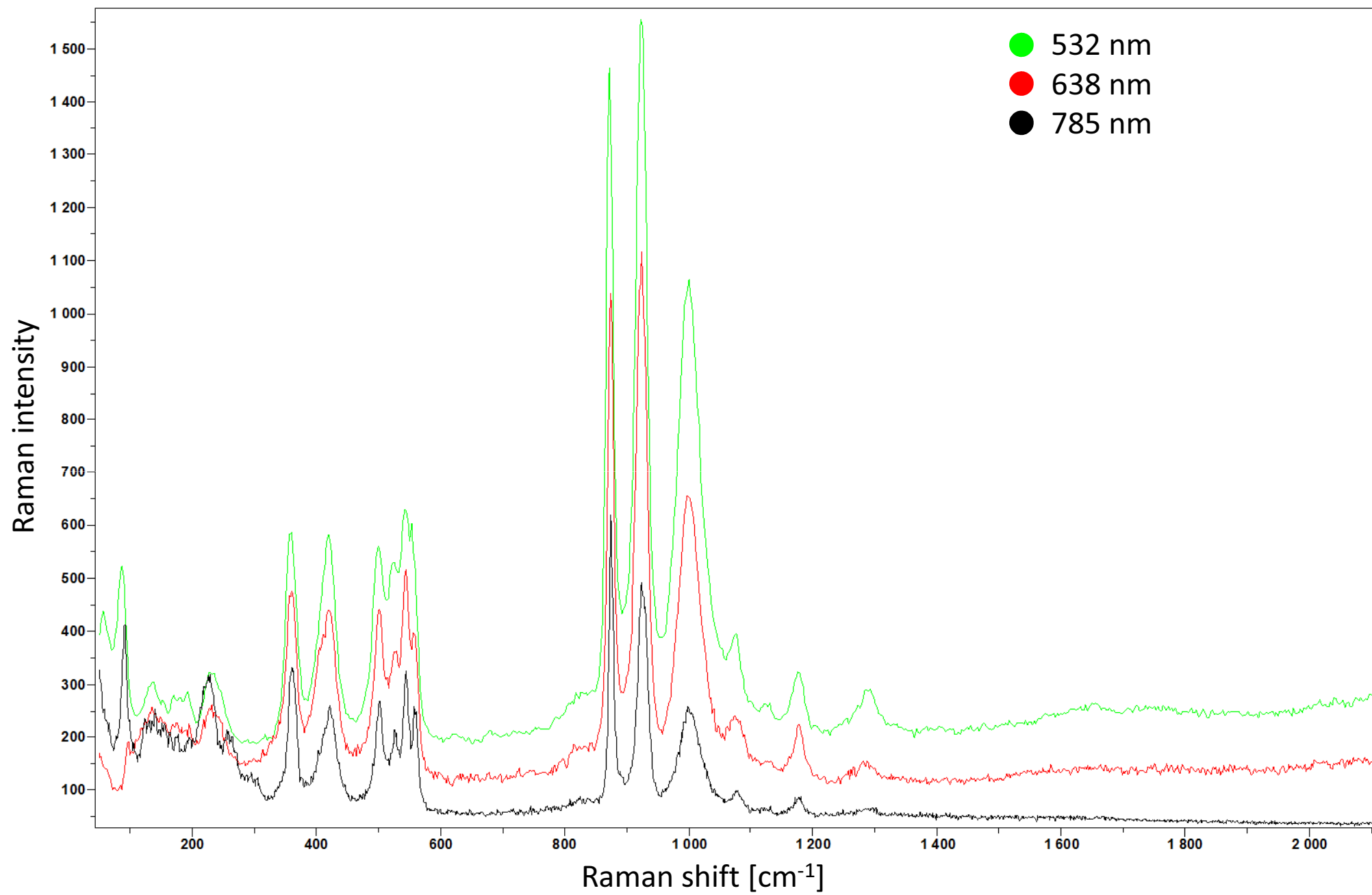

Supplement: Figure S2 — Raman spectra of sodium phosphate under three different excitation wavelengths. (PDF) [file pone.0107356.s002.pdf]

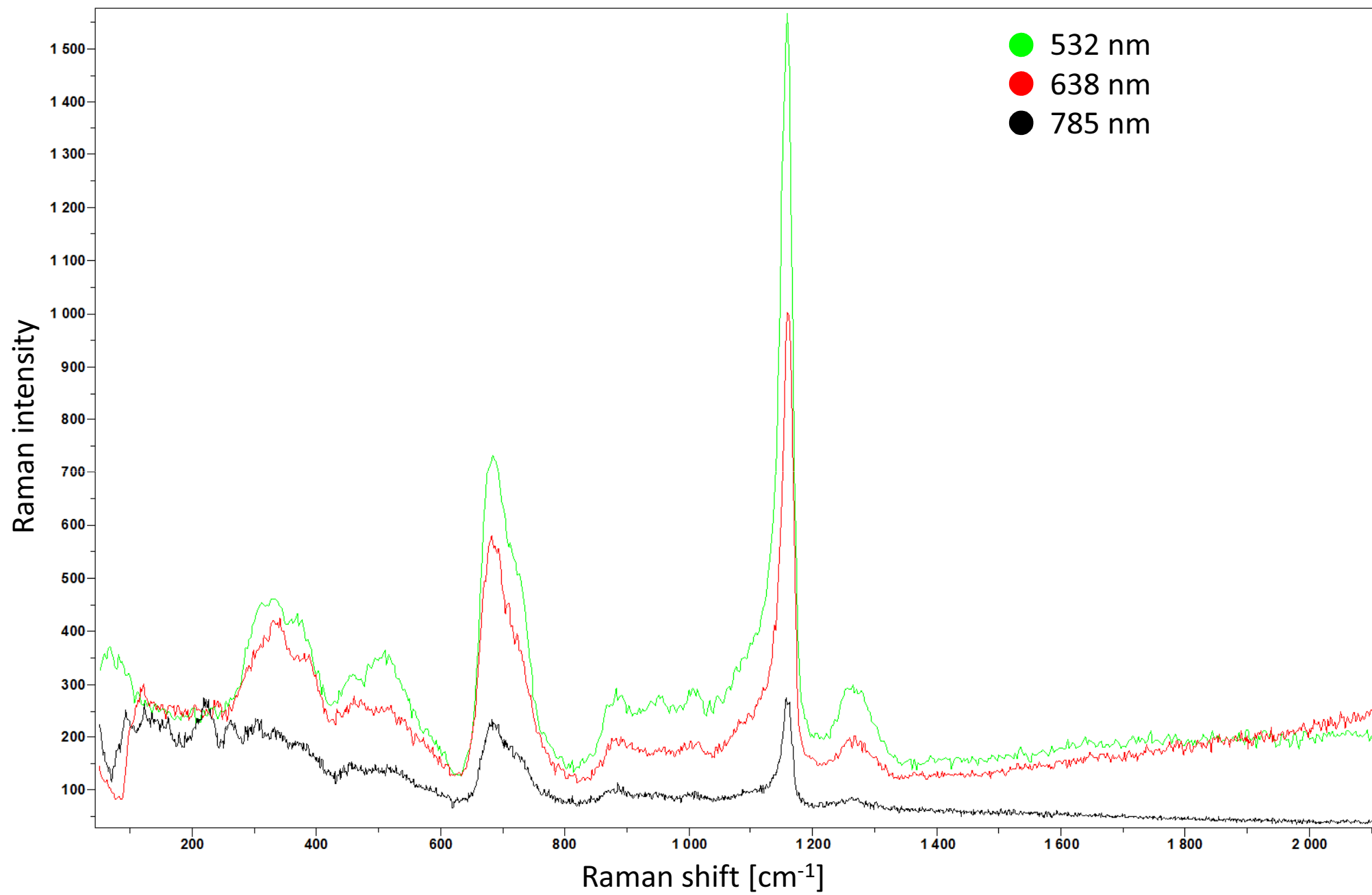

Supplement: Figure S3 — Raman spectra of sodium polyphosphate under three different excitation wavelengths. (PDF) [file pone.0107356.s003.pdf]
